# Coverage and timing of antenatal care among poor women in 6 Mesoamerican countries

**DOI:** 10.1186/s12884-016-1018-5

**Published:** 2016-08-19

**Authors:** Emily Dansereau, Claire R. McNellan, Marielle C. Gagnier, Sima S. Desai, Annie Haakenstad, Casey K. Johanns, Erin B. Palmisano, Diego Ríos-Zertuche, Alexandra Schaefer, Paola Zúñiga-Brenes, Bernardo Hernandez, Emma Iriarte, Ali H. Mokdad

**Affiliations:** 1 Institute for Health Metrics and Evaluation, 2301 5th Ave, Suite 600, Seattle, WA USA; 2 Harvard School of Public Health, 677 Huntington Ave., Boston, MA USA; 3 Salud Mesoamérica Initiative/Inter-American Development Bank, Calle 50, Edificio Tower Financial Center (Towerbank), Piso 23, Panamá, Panamá

**Keywords:** Antenatal care, Prenatal care, Coverage, Salud Mesoamérica Initiative, Skilled ANC, Household surveys

## Abstract

**Background:**

Poor women in the developing world have a heightened need for antenatal care (ANC) but are often the least likely to attend it. This study examines factors associated with the number and timing of ANC visits for poor women in Guatemala, Honduras, Mexico, Nicaragua, Panama, and El Salvador.

**Methods:**

We surveyed 8366 women regarding the ANC they attended for their most recent birth in the past two years. We conducted logistic regressions to examine demographic, household, and health characteristics associated with attending at least one skilled ANC visit, four skilled visits, and a skilled visit in the first trimester.

**Results:**

Across countries, 78 % of women attended at least one skilled ANC visit, 62 % attended at least four skilled visits, and 56 % attended a skilled visit in the first trimester. The proportion of women attending four skilled visits was highest in Nicaragua (81 %) and lowest in Guatemala (18 %) and Panama (38 %). In multiple countries, women who were unmarried, less-educated, adolescent, indigenous, had not wanted to conceive, and lacked media exposure were less likely to meet international ANC guidelines. In countries with health insurance programs, coverage was associated with attending skilled ANC, but not the timeliness.

**Conclusions:**

Despite significant policy reforms and initiatives targeting the poor, many women living in the poorest regions of Mesoamérica are not meeting ANC guidelines. Both supply and demand interventions are needed to prioritize vulnerable groups, reduce unplanned pregnancies, and reach populations not exposed to common forms of media. Top performing municipalities can inform effective practices across the region.

## Background

Substantial research has demonstrated the importance of antenatal care (ANC) for improving maternal, infant, and child health outcomes [1–3]. ANC allows providers to prevent, detect, and manage obstetric complications, and is associated with a reduced risk of maternal mortality, premature birth, and low birth weight [4–6]. ANC also links women to intra- and post-partum services such as skilled birth attendance and child vaccinations [7, 8].

ANC is particularly important for poor women, who frequently face obstetric risk factors such as inadequate nutrition, limited education, and low health literacy [9]. However, while poor women have the greatest need for ANC, they are also often the least likely to attend care. Such disparities are highlighted in Latin America and the Caribbean, which has the highest income inequality of any region in the world [10]. For instance, a 2014 study found that 97.8 % of Latin American women with a secondary education attended at least one ANC visit, compared to only 74.3 % of those without education [11]. Individual countries have recorded even wider disparities, including a 2006-07 study in Nicaragua which found that 92 % of women in the wealthiest quintile attended at least four ANC visits compared to only 61 % of women in the poorest quintile [12].

Studies of ANC in low and middle income countries frequently conclude that poverty is strongly associated with a lack of ANC [11]. However, despite being identified as a priority group, much less is known about the characteristics of these poor women and the specific barriers they face. This study aims to describe ANC coverage and timing among women living in the poorest areas of six Mesoamerican countries. We assess the variation in coverage among these poor populations, and identify demographic and household factors associated with ANC care that can be used to develop and target interventions to reduce health disparities.

## Methods

### Study setting and sample

This study uses baseline data collected for the Salud Mesoamérica Initiative (SMI), an intervention to address health issues faced by the poorest populations in eight Mesoamerican countries. For this analysis, we used the household and women’s survey data from Guatemala, Honduras, Mexico (exclusively in the state of Chiapas), Nicaragua, Panama, and El Salvador. We excluded Belize and Costa Rica from this analysis, as these countries’ surveys did not contain the necessary information about antenatal care.

In each country, SMI selected the municipalities or areas in the poorest wealth quintile. We randomly selected census segments in these areas using probability proportional to size. We conducted our own census of households in the selected segments and randomly selected households to participate in the survey. Within selected households, all women aged 15–49 were asked to complete a maternal health questionnaire. The women’s response rate was 95 %. The sample size in each country was based on a country-specific power calculation for detecting changes in health indicators under the results-based financing scheme employed by SMI.

### Data collection

The SMI baseline surveys were conducted from March 1, 2011 to August 31, 2013. Data were collected electronically by trained interviewers using computer‐assisted personal interviewing (CAPI) software, and continuously uploaded to a secure database. This process allowed for timely and systematic quality monitoring by researchers in the United States. Additional details on SMI methodology and implementation are available elsewhere [13].

The household survey had several modules. The first was a household questionnaire capturing information on assets, wealth, and characteristics of the home. The second was a maternal health questionnaire collecting demographic, health behavior, and reproductive health information on women aged 15–49 years. We used information from each woman’s most recent birth, and restricted our sample to births occurring in the two years before the survey to minimize recall bias. Women reported if they had attended antenatal care, the total number of visits, and how many months they had been pregnant when they attended their first ANC visit. They also reported the type of provider they saw at each visit.

### Analysis

#### Outcome variables

Prior research has found that different factors are associated with attending at least one ANC and returning for multiple visits [11]. Therefore, we examined three distinct outcomes: (1) attending at least one skilled ANC visit, (2) attending at least four skilled visits, and (3) the timing of the first skilled visit. We calculated these outcomes for each country and municipality, to compare them across and within countries.

The first outcome of interest was a binary indicator of whether the woman attended at least one skilled ANC visit. We classified the types of providers seen at each visit into skilled and unskilled. Skilled providers included doctors and professional nurses. Unskilled providers included unskilled midwives, auxiliary nurses without a university degree, community health workers (CHW), lab techs, pharmacy assistants, traditional healers, relatives, and others. A woman was defined as attending at least one skilled ANC visit if she had one or more visits with a skilled provider.

The second outcome of interest was a binary indicator of whether the woman attended at least four skilled ANC visits, as recommended by the World Health Organization (WHO). We examined this outcome only among women who attended at least one skilled ANC, to isolate the issues of attending at least one visit and attending ongoing care. A woman was defined as attending at least four skilled ANC visits if she had four or more visits with a skilled provider. The questions about provider type were asked differently in Guatemala, where women reported the type of provider seen at their first ANC visit and the type of provider they usually saw for ANC. In these cases, we assumed that they saw the usual provider at all subsequent visits. It was not possible to calculate the number of skilled visits in El Salvador as the survey did not contain the necessary information.

The third outcome of interest was a binary indicator of whether the woman had attended a skilled ANC visit in the first trimester. This is recommended by the WHO and all six countries in the analysis. We examined this outcome only among women who attended at least one skilled ANC visit, to isolate the issues of attending at least one visit, and the timing of care. Women reported the month of their first visit, which we classified into trimesters. They did not report the timing of any subsequent visits. Among those attending at least one skilled ANC, 95 % saw a skilled provider at their first visit, meaning that the month of their first visit was also the month of their first skilled visit. However, we were unable to determine the timing of the first skilled visit for the 5 % of women whose first visit was with an unskilled provider, but later saw a skilled provider, because the timing of visits after the first unskilled visit was not recorded. These women were excluded from our analysis of visit timing.

### Independent variables

Demographic variables included the woman’s marital status, parity, age, occupation, and education.

Household characteristics included head of household gender, rural or urban location, whether someone in the household spoke an indigenous language, ownership of a cellular phone, and monthly household expenditure per capita.

We also included health characteristics, including the woman’s self-report of whether she wanted to conceive this child or not; whether she reported any heavy vaginal bleeding during the pregnancy in question; whether she had ever experienced a past obstetric complication (still birth or miscarriage); whether she had received counseling from a CHW in the month prior to survey; media exposure in the week prior to survey (newspaper, radio, or television); satisfaction with her most recent health facility visit (among those with a recent visit); and travel time to the usual health facility. If travel time to the usual health facility was missing, we used the travel time to the closest health facility. If that too was missing, we used the median travel time to the usual or closest facility among households in that segment. In Mexico and Guatemala, we included an indicator of whether the woman had health insurance. Participation in the Oportunidades (now called Prospera) conditional cash transfer program was also an independent variable in Mexico.

### Regression analysis

We used country-specific logistic regression models to examine the demographic, household, and health factors associated with each of the three binary outcomes. We used the svy commands in Stata 13.1 to account for the sampling scheme and potential clustering of women within segments. All estimates were computed using survey weights, unless otherwise noted.

## Results

We collected data from 8366 women giving birth in the past two years in El Salvador, Guatemala, Honduras, Mexico, Nicaragua, and Panama (Table 1). Approximately 15 % of women in our study were under 20 years old and the average number of total children ranged from 2.5 in Nicaragua to 3.5 in Panama. The majority were married (84 %) and homemakers (91 %). The percent of sampled women with no education ranged from 6 % in Honduras to 30 % in Guatemala. Women lived in a home with a female head of household 16 % of the time, ranging from 8 % in Mexico to 26 % in in Nicaragua and El Salvador. Most households (76 %) were located in a rural area. On average, households spent $33.13 per capita per month.

**Table 1:** Demographic, household and health characteristics of sampled women

	Guatemala	Honduras	Mexico	Nicaragua	Panama	El Salvador	Total
N	1757	1326	2193	625	1079	1386	8366
Demographics							
Married	86 %	80 %	91 %	77 %	81 %	77 %	84 %
Mean number of children	3.1	2.7	3.1	2.5	3.5	2.7	3.0
Age							
< 20 years	16 %	16 %	13 %	15 %	15 %	16 %	15 %
20–34	65 %	69 %	70 %	72 %	61 %	70 %	68 %
> =35 years	19 %	15 %	18 %	14 %	24 %	14 %	17 %
Occupation							
Employed, working for money	4 %	8 %	6 %	13 %	6 %	8 %	7 %
Homemaker	93 %	89 %	93 %	84 %	91 %	90 %	91 %
Other	3 %	3 %	2 %	3 %	3 %	2 %	2 %
Literate	41 %	66 %	58 %	77 %	62 %	80 %	60 %
Education							
Pre-primary/none	30 %	6 %	17 %	9 %	15 %	10 %	17 %
Primary	52 %	71 %	50 %	46 %	55 %	54 %	54 %
Post-primary	18 %	23 %	33 %	45 %	31 %	37 %	29 %
Household characteristics							
Female head of household	13 %	19 %	8 %	26 %	25 %	26 %	16 %
Urban	15 %	15 %	38 %	32 %	0 %	28 %	24 %
Languages spoken in household							
Only Spanish	27 %	100 %	30 %	90 %	3 %	unmeasured	44 %
Spanish and Indigenous	68 %	0 %	64 %	10 %	67 %		49 %
Only Indigenous	5 %	0 %	7 %	0 %	30 %		6 %
Has cell phone	74 %	75 %	37 %	68 %	52 %	82 %	62 %
Monthly exp. per capita (USD)	$28.09	$39.07	$33.65	$35.71	$33.26	$33.54	$33.13
Health characteristics							
Wanted pregnancy	87 %	72 %	80 %	67 %	70 %	72 %	78 %
Vaginal bleeding	16 %	9 %	12 %	7 %	38 %	7 %	13 %
Previous obstetric complications	10 %	11 %	9 %	10 %	6 %	10 %	9 %
CHW counseling in prior month	7 %	11 %	17 %	2 %	6 %	40 %	14 %
Media exposure in prior month	67 %	82 %	67 %	90 %	60 %	87 %	74 %
Satisfied with last medical visit^a^	91 %	95 %	86 %	93 %	87 %	unmeasured	90 %
Travel time to usual facility							
< 15 min	29 %	34 %	34 %	23 %	61 %	20 %	32 %
15 to <30 min	27 %	26 %	27 %	26 %	7 %	28 %	26 %
30 to <60 min	22 %	18 %	23 %	21 %	9 %	28 %	22 %
60+ min	21 %	22 %	15 %	30 %	23 %	24 %	21 %
Insured	11 %	1 %	83 %	4 %	5 %	8 %	31 %
Oportunidades	N/A	N/A	63 %	N/A	N/A	N/A	N/A

^a^among those with recent visit

Insurance coverage was highest in Mexico (83 %), Guatemala (11 %), and El Salvador (8 %). Among those insured in Mexico, the vast majority (95 %) were insured by Seguro Popular. Approximately 9 % of women reported experiencing previous obstetric complications (a past stillbirth or miscarriage) and 13 % had heavy vaginal bleeding during the pregnancy of interest. Salvadoran women were most likely to have received CHW counseling in the past month (40 %), and Nicaraguans had the most media exposure in this period (90 % with any exposure to TV, radio, or newspaper). Nearly a third (32 %) of women lived within 15 min of their usual health facility, while over a fifth (21 %) were more than an hour away.

### Women attending at least one ANC visit

**Fig. 1:**
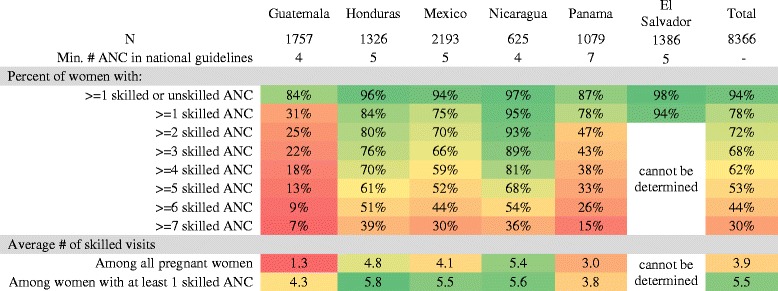
Heatmap showing the number of ANC visits by country. Note: Skilled ANC includes visits with doctors and professional nurses. Unskilled ANC includes visits with unskilled midwives, auxiliary nurses without a university degree, community health workers, lab techs, pharmacy assistants, traditional healers, relatives, and others. It was not possible to calculate the number of skilled visits in El Salvador as the survey did not contain the necessary information

Approximately 94 % of women attended at least one skilled or unskilled ANC visit during their previous pregnancy, while 78 % attended at least one ANC visit with a skilled attendant (Fig. 1). The gap between any ANC and skilled ANC was largest in Guatemala (84 % attending any and 31 % attending skilled) and Mexico (94 % attending any and 75 % attending skilled). In Guatemala, this was largely explained by the large proportion of women attending care with auxiliary nurses, while in Mexico, the gap was primarily due to women attending care with unskilled midwives. Guatemala had the lowest percent of women attending at least one skilled ANC visit (31 %), while Nicaragua had the highest (95 %). All countries except Guatemala and Panama (in which we visited only two comarcas, rather than municipalities) had at least one municipality where 100 % of women attended at least one skilled ANC visit. Coverage of at least one skilled ANC exceeded 90 % in all Nicaraguan municipalities, while Mexico, Honduras, and Guatemala all had municipalities where less than half of women attended at least one skilled ANC.

From this point forward, all results refer only to skilled ANC visits.

### Number of skilled ANC visits

Guatemala and Panama had the lowest average number of skilled ANC visits both among all pregnant women (1.3 in Guatemala, 3.0 in Panama), and among women who attended at least one skilled ANC visit (4.3 in Guatemala, 3.8 in Panama) (Fig. 1). Nicaragua had the greatest average number of skilled visits among all pregnant women (5.4), while Honduras had the most among women who attended at least one skilled ANC visit (5.8). All countries had at least one municipality where the average number of skilled visits among those attending at least one skilled ANC exceeded five. However, Mexico and Guatemala also had municipalities where the average was fewer than three.

Overall, 62 % percent of women met the WHO standard of at least four skilled ANC visits, ranging from 18 % in Guatemala to 81 % in Nicaragua (Fig. 1). Honduras, Mexico, and Nicaragua contained municipalities where over 90 % of women had at least four skilled visits, but Honduras and Mexico also contained municipalities where less than 40 % of women met the threshold. In every municipality we surveyed in Guatemala, less than half of women attended four skilled ANC visits.

Country standards for the minimum number of visits were met by 15 % of women in Panama (7 visits); 18 % in Guatemala (4 visits); 52 % in Mexico (5 visits); 61 % in Honduras (5 visits); and 81 % in Nicaragua (4 visits) [14–18].

### Timing of first skilled ANC visit

**Fig. 2:**
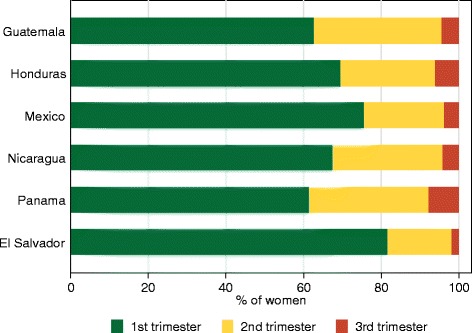
Trimester of first skilled ANC visit, among women receiving at least one skilled ANC

Across countries, 56 % of all pregnant women reported a skilled ANC visit during the first trimester, ranging from 18 % in Guatemala to 74 % in El Salvador. Among women who attended at least one skilled ANC, 73 % had a skilled visit in the first trimester, from a low of 63 % in Panama and Guatemala to a high of 82 % in El Salvador (Fig. 2). The proportion of women whose first skilled visit did not occur until the third trimester ranged from 2 % in El Salvador to 7 % in Panama. Guatemala, Honduras, Mexico, and Nicaragua all had a municipality where 100 % of women attended skilled ANC in the first trimester. However, all countries but Panama contained a municipality where less than half of women attended skilled care within the first trimester.

### Factors associated with attending at least one skilled ANC visit

Compared to women with no education, a post-primary education was significantly associated with an increased odds of attending at least one skilled ANC visit in Guatemala (Odds Ratio [OR] = 2.92, 95 % Confidence Interval [CI] = 2.03-4.21), Honduras (OR = 4.50, CI = 1.57-12.87), Mexico (OR = 2.58, CI = 1.72-3.89), Nicaragua (OR = 6.68, CI = 2.04-21.86), and Panama (OR = 2.24, CI = 1.02-4.89) (Table 2). Being in the wealthiest quintile of women surveyed was also significantly associated with increased odds in Guatemala (OR = 1.59, CI = 1.02-2.47), Honduras (OR = 3.35, CI = 1.52-7.37), and Mexico (OR = 2.47, CI = 1.52-4.02).

**Table 2:** Logistic regression results for factors associated with attending at least one skilled ANC visit

	Guatemala		Honduras		Mexico		Nicaragua		Panama		El Salvador	
Married	0.91	[0.64,1.30]	1.08	[0.66,1.79]	1.10	[0.69,1.74]	0.70	[0.20,2.52]	0.71	[0.37,1.37]	0.97	[0.39,2.41]
Parity (ref: 1st child)												
2nd-3rd child	0.61**	**[0.46,0.82]**	1.10	[0.59,2.06]	1.30	[0.87,1.93]	0.57	[0.21,1.58]	1.18	[0.46,3.04]	0.73	[0.26,2.04]
4th-5th child	0.62*	**[0.42,0.91]**	0.90	[0.46,1.76]	0.80	[0.54,1.19]	0.62	[0.16,2.35]	1.07	[0.41,2.79]	0.18**	**[0.05,0.62]**
6th + child	1.06	[0.68,1.67]	0.63	[0.27,1.49]	0.76	[0.49,1.19]	0.24	[0.04,1.38]	0.97	[0.39,2.39]	0.40	[0.07,2.14]
Age (ref: <20)												
20–34	2.41***	**[1.66,3.49]**	0.96	[0.49,1.89]	1.11	[0.74,1.65]	1.21	[0.33,4.49]	0.86	[0.36,2.05]	1.69	[0.47,6.08]
> =35	2.42***	**[1.52,3.87]**	1.25	[0.47,3.38]	1.45	[0.88,2.39]	1.41	[0.32,6.27]	2.43	[0.89,6.69]	1.82	[0.35,9.56]
Education (ref: None)												
Primary	1.89***	**[1.41,2.54]**	1.86	[0.89,3.88]	1.53**	**[1.13,2.07]**	2.50	[1.00,6.28]	1.92	[1.00,3.70]	1.84	[0.63,5.34]
Post-primary	2.92***	**[2.03,4.21]**	4.50**	**[1.57,12.87]**	2.58***	**[1.72,3.89]**	6.68**	**[2.04,21.86]**	2.24*	**[1.02,4.89]**	1.27	[0.31,5.24]
Female head of household	0.90	[0.61,1.32]	1.02	[0.62,1.70]	1.39	[0.86,2.27]	0.60	[0.24,1.52]	1.30	[0.68,2.47]	1.66	[0.62,4.45]
Urban	2.07***	**[1.39,3.09]**	1.40	[0.68,2.89]	1.17	[0.79,1.72]	1.78	[0.29,10.87]	(all rural)		0.99	[0.37,2.67]
Relative household expenditure per capita (ref: 1st quintile)												
2nd quintile	0.96	[0.62,1.49]	0.88	[0.50,1.53]	1.19	[0.85,1.67]	0.49	[0.17,1.41]	1.14	[0.36,3.65]	2.26	[0.83,6.13]
3rd quintile	1.15	[0.72,1.84]	0.75	[0.39,1.45]	1.27	[0.87,1.84]	0.98	[0.25,3.78]	1.01	[0.35,2.90]	1.35	[0.52,3.56]
4th quintile	0.97	[0.64,1.45]	1.31	[0.73,2.37]	1.49*	[1.05,2.11]	0.31	[0.08,1.23]	0.80	[0.25,2.58]	2.83	[0.96,8.38]
5th quintile	1.59*	**[1.02,2.47]**	3.35**	**[1.52,7.37]**	2.47***	**[1.52,4.02]**	0.39	[0.10,1.56]	0.87	[0.27,2.87]	2.96	[0.88,9.97]
Owns mobile phone	1.37*	**[1.04,1.81]**	1.66*	**[1.07,2.56]**	1.33	[0.93,1.91]	0.93	[0.36,2.42]	1.31	[0.75,2.29]	1.77	[0.79,3.98]
Pregnancy was wanted	1.03	[0.73,1.47]	0.84	[0.51,1.39]	0.95	[0.68,1.31]	1.62	[0.72,3.62]			0.86	[0.34,2.13]
Reported vaginal bleeding	1.5	[0.91,2.49]	0.67	[0.33,1.39]	1.37	[0.91,2.07]	1	[1.00,1.00]	0.62	[0.30,1.30]	0.61	[0.16,2.33]
Previous complications	1	[0.71,1.41]	1.28	[0.75,2.19]	0.89	[0.58,1.37]	1.19	[0.40,3.49]	0.97	[0.40,2.35]	1.94	[0.50,7.57]
CHW in the past month	0.75	[0.47,1.20]	1.64	[0.86,3.11]	1.44	[0.98,2.10]	0.34	[0.03,3.71]	0.7	[0.25,1.92]	1.37	[0.59,3.18]
Media in the past week	1.65**	**[1.20,2.27]**	0.56*	**[0.32,0.97]**	0.91	[0.70,1.17]	1.22	[0.49,3.06]	1.21	[0.70,2.08]	0.68	[0.23,2.01]
Time to health facility (ref: <15 min)												
15–<30 min	1.11	[0.80,1.54]	0.9	[0.46,1.77]	1.21	[0.86,1.70]	0.34	[0.06,1.81]	0.77	[0.29,2.08]	0.19*	**[0.04,0.87]**
30–<60 min	1.26	[0.89,1.79]	0.83	[0.45,1.50]	1.11	[0.74,1.64]	0.36	[0.07,1.92]	1.12	[0.41,3.07]	0.23	[0.05,1.15]
60+ minutes	0.81	[0.54,1.22]	0.81	[0.46,1.43]	1.29	[0.74,2.25]	0.29	[0.05,1.49]	0.46	[0.20,1.08]	0.22	[0.04,1.09]
Insured	1.60*	**[1.09,2.36]**	0.83	[0.07,9.69]	1.92***	**[1.43,2.57]**	0.86	[0.08,8.83]	0.94	[0.29,3.10]	1.08	[0.20,5.75]
Languages spoken in household (ref: Spanish only)												
Spanish and indigenous	0.97	[0.73,1.30]			0.42***	**[0.27,0.65]**	0.46	[0.07,3.05]	1.64	[0.22,12.26]		
Indigenous only	0.7	[0.37,1.33]			0.33***	**[0.18,0.61]**			1.28	[0.16,10.30]		
Oportunidades					1.52**	**[1.12,2.07]**						

Bold text indicates results that are statistically significant in terms of the following: *p < .05; **p < .01; ***p < .001

In Guatemala and Mexico, the countries with the highest insurance coverage, having insurance was associated with increased odds of attending at least one skilled visit (Guatemala: OR = 1.60, CI = 1.09-2.36; Mexico: OR = 1.92, CI = 1.43-2.57). In Mexico, participating in Oportunidades was also associated with increased odds (OR = 1.52, CI = 1.12-2.07), while living in a household that spoke both Spanish and an indigenous language (OR = 0.42, CI = 0.27-0.65) or only an indigenous language (OR = 0.33, CI = 0.18-0.61) was associated with lower odds, compared to households speaking only Spanish.

### Factors associated with receiving four skilled ANC visits, among those with at least one skilled ANC

Compared to women with no education, those with a post-primary education had increased odds of attending four skilled ANC visits in Guatemala (OR = 2.18, CI = 1.10-4.31), Mexico (OR = 1.99, CI = 1.25-3.17), and Nicaragua (OR = 3.89, CI = 1.49-10.13) (Table 3). Recent exposure to TV, radio or the newspaper was associated with increased odds of four skilled visits in Guatemala (OR = 1.63, CI = 1.05-2.53), Mexico (OR = 1.38, CI = 1.01-1.87), Nicaragua (OR = 2.14, CI = 1.15-3.98), and Panama (OR = 2.18, CI = 1.29-3.69). In Nicaragua, lower parity and greater age were associated with greater odds of four skilled visits.

**Table 3:** Logistic regression results for factors associated with attending four skilled ANC visits, among women attending at least one skilled ANC visit

	Guatemala		Honduras		Mexico		Nicaragua		Panama	
Married	0.92	[0.54,1.58]	1.48	[0.85,2.58]	1.22	[0.70,2.13]	1.14	[0.65,1.99]	1.23	[0.68,2.22]
Parity (ref: 1st child)										
2nd–3rd child	1.38	[0.85,2.25]	0.74	[0.40,1.36]	1.20	[0.82,1.76]	0.31**	**[0.15,0.64]**	1.50	[0.68,3.29]
4th–5th child	1.18	[0.64,2.17]	0.55	[0.27,1.12]	0.97	[0.57,1.64]	0.28**	**[0.12,0.66]**	1.22	[0.44,3.37]
6th + child	1.25	[0.54,2.88]	0.30**	**[0.13,0.67]**	0.74	[0.43,1.25]	0.23*	**[0.07,0.78]**	1.25	[0.49,3.20]
Age (ref: <20)										
20–34	0.85	[0.44,1.64]	1.45	[0.73,2.84]	1.42	[0.88,2.29]	3.20*	**[1.32,7.74]**	0.71	[0.29,1.75]
> =35	1.42	[0.61,3.31]	1.69	[0.69,4.11]	1.67	[0.91,3.07]	3.94*	**[1.29,12.03]**	0.44	[0.14,1.44]
Education (ref: None)										
Primary	1.55	[0.84,2.88]	1.40	[0.64,3.09]	1.35	[0.91,2.01]	2.51*	**[1.22,5.16]**	0.95	[0.42,2.12]
Post-primary	2.18*	**[1.10,4.31]**	1.78	[0.71,4.44]	1.99**	**[1.25,3.17]**	3.89**	**[1.49,10.13]**	0.87	[0.37,2.07]
Female head of household	0.78	[0.41,1.48]	1.02	[0.52,1.98]	0.90	[0.55,1.49]	1.25	[0.70,2.23]	1.45	[0.94,2.22]
Urban	1.57	[0.87,2.85]	0.81	[0.50,1.34]	1.27	[0.87,1.85]	1.40	[0.64,3.05]	(all rural)	
Relative household expenditure per capita (ref: 1st quintile)										
2nd quintile	0.91	[0.43,1.89]	1.08	[0.56,2.05]	1.19	[0.81,1.76]	0.71	[0.33,1.51]	3.67***	**[1.79,7.53]**
3rd quintile	0.87	[0.46,1.64]	1.46	[0.80,2.66]	1.18	[0.76,1.82]	0.60	[0.28,1.29]	1.25	[0.63,2.48]
4th quintile	0.83	[0.47,1.48]	1.18	[0.64,2.18]	1.10	[0.68,1.76]	0.88	[0.33,2.31]	1.91	[0.91,4.02]
5th quintile	0.84	[0.46,1.55]	1.12	[0.57,2.23]	1.39	[0.81,2.38]	0.48	[0.20,1.16]	3.09**	**[1.38,6.90]**
Owns mobile phone	0.85	[0.54,1.33]	0.78	[0.48,1.28]	0.94	[0.66,1.33]	1.08	[0.65,1.78]	1.20	[0.67,2.15]
Pregnancy was wanted	2.46**	**[1.39,4.33]**	1.18	[0.76,1.82]	1.32	[0.94,1.85]	1.53	[0.88,2.64]		
Reported vaginal bleeding	0.72	[0.37,1.38]	1.39	[0.58,3.32]	0.82	[0.50,1.36]	1.02	[0.41,2.53]	1.69	[0.73,3.94]
Previous complications	1.44	[0.76,2.70]	0.83	[0.44,1.56]	0.62*	**[0.40,0.97]**	1.33	[0.55,3.24]	1.77	[0.55,5.64]
CHW in the past month	0.68	[0.30,1.50]	2.37	[0.83,6.76]	1.16	[0.80,1.68]	1.20	[0.16,8.74]	1.20	[0.38,3.77]
Media in the past week	1.63*	**[1.05,2.53]**	1.31	[0.81,2.13]	1.38*	**[1.01,1.87]**	2.14*	**[1.15,3.98]**	2.18**	**[1.29,3.69]**
Time to health facility (ref: <15 min)										
15–<30 min	0.68	[0.42,1.09]	0.72	[0.40,1.30]	1.06	[0.75,1.51]	1.21	[0.45,3.22]	1.42	[0.45,4.50]
30–<60 min	1.16	[0.63,2.16]	0.66	[0.37,1.17]	1.07	[0.74,1.56]	0.88	[0.39,1.97]	0.77	[0.35,1.72]
60+ minutes	0.69	[0.36,1.33]	0.69	[0.38,1.26]	0.98	[0.58,1.66]	0.72	[0.33,1.59]	0.71	[0.35,1.43]
Insured	1.78	[0.92,3.44]	1.21	[0.23,6.55]	1.55*	**[1.10,2.20]**	1.65	[0.39,7.07]	1.22	[0.34,4.32]
Languages spoken in household (ref: Spanish only)										
Spanish and indigenous	1.08	[0.66,1.76]			0.54**	**[0.37,0.78]**	0.55	[0.22,1.39]	0.35	[0.08,1.49]
Indigenous only	0.80	[0.25,2.59]			0.47*	**[0.26,0.85]**			0.41	[0.09,1.88]
Oportunidades					1.31	[0.93,1.83]				

Bold text indicates results that are statistically significant in terms of the following: *p < .05; **p < .01; ***p < .001

Note: In El Salvador, it was not possible to determine whether a woman attended four skilled ANC visits due to the design of the survey

Compared to those without insurance, insurance coverage was associated with increased odds of four skilled visits in Mexico (OR = 1.55, CI = 1.10-2.20), while participation in Oportunidades did not significantly increase the odds of four skilled visits. In Mexico, a gradient was again observed in which households speaking both Spanish and an indigenous language (OR = 0.54, CI = 0.37-0.78) or only an indigenous language (OR = 0.47, CI = 0.26-0.85) had lower odds of attending four skilled visits compared to those speaking Spanish only.

### Factors associated with attending skilled ANC in the first trimester, among those with at least one skilled ANC visit

Compared to women with no education, post-primary education was associated with increased odds of attending skilled ANC in the first trimester in Guatemala (OR = 3.08, CI = 1.42-6.68), Mexico (OR = 1.84, CI = 1.15-2.94), and Nicaragua (OR = 2.30, CI = 1.07-4.96) (Table 4). Having more children was associated with lower odds of a first trimester skilled visit in Honduras, Nicaragua, and El Salvador. Being married was significantly associated with increased odds of first trimester skilled ANC in Mexico (OR = 2.29, CI = 1.42-3.70) and El Salvador (OR = 2.04, CI = 1.12-3.71). Women who wanted to conceive had increased odds of a first trimester skilled visit in Guatemala (OR = 2.85, CI = 1.48-5.49) and Nicaragua (OR = 1.54, CI = 1.01-2.36), while those who had previously experienced an obstetric complication had increased odds in Honduras (OR = 2.29, CI = 1.14-4.57) and Nicaragua (OR = 1.91, CI = 1.11-3.30). Living over an hour away from the usual health facility was associated with lower odds of first trimester skilled ANC in Nicaragua (OR = 0.46, CI = 0.27-0.79) and Panama (OR = 0.43, CI = 0.22-0.84). Households speaking indigenous languages had lower odds in Mexico and Nicaragua. Insurance status was not associated with attending skilled ANC in the first trimester in any country, nor was participation in Oportunidades in Mexico.

**Table 4:** Logistic regression results for factors associated with attending a skilled ANC visit in the first trimester, among women attending at least one skilled ANC visit

	Guatemala		Honduras		Mexico		Nicaragua		Panama		El Salvador	
Married	0.66	[0.35,1.23]	1.78	[0.97,3.26]	2.29***	**[1.42,3.70]**	1.60	[1.00,2.55]	0.75	[0.40,1.43]	2.04*	**[1.12,3.71]**
Parity (ref: 1st child)												
2nd–3rd child	1.18	[0.68,2.05]	0.61*	**[0.37,1.00]**	0.73	[0.47,1.11]	0.59*	**[0.37,0.94]**	1.04	[0.46,2.34]	0.74	[0.41,1.32]
4th–5th child	0.99	[0.50,1.97]	0.48*	**[0.24,0.98]**	0.66	[0.38,1.15]	0.42*	**[0.19,0.89]**	0.63	[0.22,1.84]	0.46	[0.19,1.11]
6th + child	1.29	[0.47,3.51]	0.29**	**[0.14,0.62]**	0.69	[0.39,1.24]	0.37*	**[0.14,0.98]**	0.39	[0.11,1.42]	0.27*	**[0.10,0.73]**
Age (ref: <20)	1.00	[1.00,1.00]	1.00	[1.00,1.00]	1.00	[1.00,1.00]	1.00	[1.00,1.00]	1.00	[1.00,1.00]	1.00	[1.00,1.00]
20–34	1.33	[0.72,2.46]	1.68	[0.87,3.24]	2.57***	**[1.67,3.97]**	1.78	[0.92,3.45]	1.42	[0.68,2.97]	1.67	[0.82,3.39]
> =35	1.93	[0.78,4.77]	1.28	[0.56,2.93]	3.16***	**[1.69,5.90]**	1.39	[0.56,3.44]	1.62	[0.52,5.07]	1.73	[0.67,4.50]
Education (ref: None)												
Primary	2.04*	**[1.11,3.74]**	2.17	[0.94,5.02]	1.35	[0.90,2.03]	1.52	[0.78,2.96]	0.65	[0.31,1.35]	0.40	[0.15,1.05]
Post-primary	3.08**	**[1.42,6.68]**	1.87	[0.70,5.03]	1.84*	**[1.15,2.94]**	2.30*	**[1.07,4.96]**	0.74	[0.35,1.59]	0.59	[0.19,1.84]
Female head of household	0.96	[0.47,1.97]	1.15	[0.59,2.23]	1.10	[0.70,1.72]	1.23	[0.71,2.10]	1.48	[0.80,2.75]	0.81	[0.46,1.42]
Urban	0.93	[0.52,1.66]	0.69	[0.42,1.13]	1.10	[0.78,1.57]	1.48	[0.82,2.67]	(all rural)		0.57	[0.31,1.07]
Relative household expenditure per capita (ref: 1st quintile)												
2nd quintile	0.54	[0.22,1.36]	2.01**	**[1.24,3.27]**	1.07	[0.66,1.74]	0.84	[0.46,1.55]	0.98	[0.39,2.46]	0.95	[0.40,2.22]
3rd quintile	0.69	[0.29,1.65]	1.94**	**[1.18,3.19]**	0.99	[0.66,1.50]	1.07	[0.61,1.91]	0.67	[0.30,1.51]	1.68	[0.66,4.29]
4th quintile	0.92	[0.42,1.98]	1.76*	**[1.05,2.96]**	1.15	[0.72,1.84]	0.93	[0.47,1.87]	0.82	[0.27,2.44]	1.13	[0.56,2.26]
5th quintile	1.00	[0.45,2.22]	2.25**	**[1.34,3.79]**	1.33	[0.77,2.31]	1.06	[0.52,2.18]	0.85	[0.33,2.23]	0.79	[0.37,1.71]
Owns mobile phone	0.90	[0.52,1.56]	1.21	[0.76,1.93]	0.85	[0.60,1.20]	1.02	[0.70,1.46]	0.79	[0.43,1.45]	1.36	[0.72,2.55]
Pregnancy was wanted	2.85**	**[1.48,5.49]**	1.32	[0.88,2.00]	1.30	[0.96,1.75]	1.54*	**[1.01,2.36]**			1.36	[0.84,2.19]
Reported vaginal bleeding	1.07	[0.47,2.41]	1.36	[0.66,2.80]	1.37	[0.82,2.29]	0.74	[0.31,1.78]	1.37	[0.53,3.54]	1.41	[0.51,3.93]
Previous complications	1.02	[0.53,1.95]	2.29*	**[1.14,4.57]**	0.78	[0.52,1.17]	1.91*	**[1.11,3.30]**	3.82	[0.89,16.27]	1.61	[0.62,4.17]
CHW in the past month	1.73	[0.63,4.76]	1.06	[0.61,1.83]	1.26	[0.88,1.81]	1.68	[0.40,7.11]	1.01	[0.26,3.97]	0.96	[0.56,1.66]
Media in the past week	1.04	[0.61,1.75]	0.85	[0.49,1.45]	1.22	[0.90,1.66]	1.45	[0.81,2.60]	1.32	[0.75,2.33]	1.31	[0.67,2.57]
Time to health facility (ref: <15 min)												
15–<30 min	0.54*	**[0.31,0.93]**	1.08	[0.68,1.72]	0.96	[0.65,1.42]	0.72	[0.45,1.17]	0.93	[0.43,1.99]	1.13	[0.53,2.43]
30–<60 min	0.68	[0.37,1.25]	0.80	[0.45,1.43]	0.76	[0.53,1.10]	0.61	[0.34,1.07]	0.80	[0.26,2.49]	0.68	[0.30,1.53]
60+ minutes	0.68	[0.33,1.41]	1.08	[0.70,1.69]	1.48	[0.84,2.60]	0.46**	**[0.27,0.79]**	0.43*	**[0.22,0.84]**	0.75	[0.31,1.79]
Insured	1.04	[0.59,1.86]	1.58	[0.31,8.07]	1.10	[0.72,1.66]	0.91	[0.30,2.77]	1.96	[0.54,7.07]	1.20	[0.42,3.43]
Languages spoken in household (ref: Spanish only)												
Spanish and indigenous	1.01	[0.63,1.63]			0.53**	**[0.35,0.80]**	0.49*	**[0.25,0.93]**	0.94	[0.22,4.02]		
Indigenous only	0.92	[0.26,3.24]			0.41**	**[0.21,0.80]**			0.82	[0.18,3.63]		
Oportunidades					1.21	[0.88,1.68]						

Bold text indicates results that are statistically significant in terms of the following: *p < .05; **p < .01; ***p < .001

## Discussion

To our knowledge, this is the largest study of ANC coverage and timing conducted among poor women in Mesoamérica. The results reveal substantial variation in coverage across and within countries, and demonstrate that many women are not meeting ANC guidelines. While predictors of attending skilled ANC varied by country, low maternal education, adolescent pregnancies, unwanted pregnancies, speaking an indigenous language, high parity, lack of media exposure, and being unmarried were significant in multiple countries and consistent with the findings of a systematic review of ANC risk factors in developing countries [19].

Our study comes in the context of numerous efforts to reduce health disparities and achieve universal health coverage in Latin America and the Caribbean [20]. Some countries have been highly successful, such as Cuba, where 98 % of women attend four ANC visits [21]. Several countries in our study have implemented significant pro-poor programs in recent years. Nicaragua and El Salvador both underwent dramatic health reforms in the late 2000s to decrease out-of-pocket expenditures, introduce community outreach teams, and prioritize services for the poor [22, 23]. Honduras has been implementing its program for Accelerated Reduction of Maternal and Child Mortality (RAMNI) since 2008, which uses performance-based contracts for antenatal and other services in the poorest regions of the country [16]. Mexico has two well-known programs for the poor that encourage antenatal care: the Seguro Popular health insurance scheme, and the Prospera (formerly called Oportunidades) conditional cash transfer program, which directly support and incentivize ANC [24]. Despite all these efforts, universal ANC coverage has not been achieved, and a combination of demand and supply interventions are urgently needed in poor communities.

While barriers to ANC vary by country, several common findings emerge. First, countries should prioritize health services for, and seek to empower, the most vulnerable groups of women. This includes women who are unmarried, less-educated, adolescents, or indigenous. Qualitative research has identified social factors such as machismo and disempowerment as key barriers to women’s reproductive health autonomy, and many studies have established the important influence of women’s education on maternal and child health knowledge, behaviors, and outcomes [25, 26]. Women’s empowerment is also closely related to preventing unplanned pregnancies. Our study reinforces the finding that women who lack control over their fertility are less likely to know that they are pregnant, delaying their efforts to seek care [27]. This is a particular challenge in Nicaragua, where a third of women had not wanted to become pregnant, as well as in Panama, El Salvador, and Honduras. In addition to addressing the previously mentioned social factors, improving women’s access to family planning will require health system improvements. For instance, although Nicaragua’s National Health Plan prioritizes family planning, our baseline survey of health facilities for SMI found that many facilities still lacked contraceptive supplies [13]. Countries should strengthen family planning services to decrease unwanted pregnancies, and in particular should focus on programs targeting adolescents and culturally appropriate services for indigenous communities.

Another consistent finding is a strong association between media exposure and ANC attendance. This may reflect undetected differences in wealth status and living conditions, which could confound the relationship between media exposure and ANC attendance. While our finding does not draw a causal link between media messages and ANC, it does tell us that many of the women who are not attending ANC cannot be reached through mainstream media. Health programs should seek alternate forms of communication to reach these high-risk populations, such as sending health workers to communities or organizing women’s groups [28].

Additional context-specific challenges are also present in Guatemala and Panama, which had the lowest skilled ANC attendance. First, the SMI regions in these two countries, along with Mexico, contain largely indigenous populations. Guatemala’s extremely low skilled ANC rates are largely explained by women attending ANC visits with auxiliary nurses, who are not considered skilled providers. While our estimates for seeing any skilled or unskilled provider in Guatemala (84 %) were very similar to what was reported for a 2008-09 national survey (90 %), we found the proportion attending at least one visit with a skilled provider was less than one third [29]. A social network study in Guatemala also revealed that decision making about ANC is strongly influenced by women’s mothers, partners, and mothers-in-law [30]. In Panama, it is important to note that SMI is operating in two highly inaccessible regions. Therefore, it is unsurprising that our estimates of ANC coverage (87 % any ANC, 78 % at least one skilled ANC) are lower than the estimates reported for all rural women by a 2009 national survey (96 % any ANC) [31]. The geographic barriers facing women in these communities are especially influential, and will require creative solutions such as mobile teams to provide ANC. There are also supply barriers in both of these countries, and women are discouraged from attending ANC by a lack of laboratories and ultrasounds at nearby health facilities [30].

Our study has several limitations that should be considered when interpreting its findings. First, we relied on self-report by the women about their antenatal care. We minimized recall bias by restricting our analysis to births occurring in the past two years, but it will be important to compare our results with information about the timing and number of ANC visits from medical records. Additionally, the only way our study classifies “skilled” ANC is based on an international standard of provider type, and this can vary in practice by country. We did not analyze the quality of the ANC, which could imply additional efforts from countries that have already achieved high coverage of timely and continuous ANC with skilled providers. However, our study benefits from a large sample size, a current census that ensures representativeness, and a standard methodology allowing for comparison across countries.

## Conclusions

Early and ongoing ANC is crucial for ensuring the health of both mothers and children. Our findings call for prioritizing resources to vulnerable groups and designing country-specific interventions to improve ANC coverage and timing in the poorest Mesoamerican communities. While our study found large gaps in ANC coverage, we also identified many municipalities within the poorest regions of each country where most women are meeting ANC standards. This is encouraging news for public health planners, as successful municipalities may offer effective practices to improve ANC in similarly impoverished communities across the region. Qualitative studies to identify best-practices from top performing areas and better understand the factors influencing women’s choices and health system bottlenecks can help guide the development of programs.

## Abbreviations

ANC, antenatal care; CAPI, computer-assisted personal interview; CHW, community health worker; CI, confidence interval; OR, odds ratio; SMI, Salud Mesoamérica Initiative; WHO, World Health Organization
